# Spinal Langerhans cell histiocytosis with cord compression and neurological deficits: A case report

**DOI:** 10.1016/j.ijscr.2023.108351

**Published:** 2023-05-24

**Authors:** Mohammed Maan Al-Salihi, Ahmed Saleh, Muath Hussein, Alaaeldin Ahmed, Md Moshiur Rahman, Abdulnasser Alyafai

**Affiliations:** a Department of Neurosurgery, Hamad General Hospital, Doha, Qatar; b Neurosurgery Department, Holy Family Red Crescent Medical College, Dhaka, Bangladesh

**Keywords:** Histiocytosis, Langerhans cell histiocytosis, Spinal cord

## Abstract

**Introduction and importance:**

Langerhans cell histiocytosis (LCH) is a rare idiopathic disease that uncommonly affect the spine in adults.

**Case presentation:**

In this report, we presented a rare adult case of symptomatic spinal LCH with asymptomatic systemic involvement. She was a 46-year-old previously healthy lady who presented with subacute thoracic sensory level, urine retention, constipation, and pyramidal paraplegia. Her magnetic resonance imaging (MRI) of the spine revealed T6 compression fracture with an epidural mass compressing the cord.

**Clinical discussion:**

Sellar MRI showed pituitary gland enlargement with hyperintense signal in the posterior lobe. Positron emission tomography (PET)/computed tomography (CT) scan showed an increased uptake in the right parotid gland uptake and renal cortex, indicating systemic involvement.

**Conclusion:**

Surgical excision, decompression, and screw fixation were performed, and the patient improved. The prognosis is usually good in patients with solitary spinal LCH.

## Highlights

• LCH is a rare idiopathic disease that uncommonly affect the spine in adults.
• The prognosis is usually good in patients with solitary spinal LCH.
• Spontaneous resolution is seen in a considerable proportion of patients within a year.
• In focal neurological deficits, surgery may be done prior or concomitant to other treatments.

## 1 Introduction

Langerhans cell histiocytosis (LCH) is a very rare idiopathic disease characterized by abnormal proliferation of Langerhans cells, which are antigen-presenting macrophages resident mainly in the skin epidermis and lymphoid tissues [1]. The estimated incidence of LCH is approximately1 to 2 cases per million individuals per year [2]. It commonly affects infants and children below the age of 15 years, but it might also occur at any age [1].

Symptoms of LCH are highly variable based on the organs and systems involved. It can be unifocal or multifocal affecting a single organ or systems, commonly the bone, referred to as ‘eosinophilic granuloma’ [3]. It can also be multisystemic, such as Letterer-Siwe disease, affecting bone marrow, lungs, skin, liver, and spleen [4]. Rash is a common initial presentation of LCH, then dissemination to bone marrow, liver, spleen, gastrointestinal tract, lungs, lymph nodes, and pituitary gland may evolve [1].

In adults, the vast majority of cases with LCH (69 %) affect multiple systems, whereas only one third (31 %) of the cases involve a single system [5]. Lung is the most common organ affected in single system involved, constituting more than half of the cases (51 %) [5]. Bone and cutaneous involvement are the next most common systems involved in 38 % and 7 % of the cases respectively [5]. The most common bone locations affected are the skull, femur, and spine constituting 27 %, 15 %, and 6.5 %, respectively [5].

Spine LCH, despite being common in children, is very rare in adults, and only few adult cases with isolated spinal LCH were reported in the literature [5,6]. In this report, we present a rare case of thoracic spine LCH in an adult lady presenting symptomatic spinal cord compression and neurological deficits who was found to have asymptomatic pituitary and systemic involvement.

## 2 Case report

### 2.1 Case presentation

This was a 46-year-old lady with unremarkable past medical history who presented to the primary health care (PHC) on July 25th, 2022, with back pain that had been treated conservatively. Four days later, she developed with numbness of lower extremities and exacerbation of the back pain. Within one week, she returned back to the clinic as her condition was progressed to paraparesis, urine retention, and constipation. Her neurological examination revealed thoracolumbar tenderness, sensory level at T7, symmetrical lower limbs weakness of pyramidal distribution, along with brisk deep tendon jerks and Babinski sign. Her Eastern Cooperative Oncology Group (ECOG) Performance status score was 2. This case report followed the SCARE guidelines for its realization [7]***.***

### 2.2 Laboratory investigations

Her routine chemistry panel revealed a white blood count (WBC) of 10.6 thousand cells/μL, haemoglobin (Hb) level of 13.4 g/dL, a platelet count of 251 thousand cells/μL, an absolute neutrophil count (ANC) of 7.2 thousand cells/μL, an absolute lymphocyte count of 2.6 thousand cells/μL, a creatinine level of 84 mg/dL, a total bilirubin of 8.8 g/L, total proteins of 80 g/L, an albumin level of 3.9 g/L, a sodium level of 139 mEq/L, and an ionized calcium of 2.13 mg/L. The hormonal and endocrinal panel was unremarkable.

### 2.3 Radiological findings

**Fig. 1:**
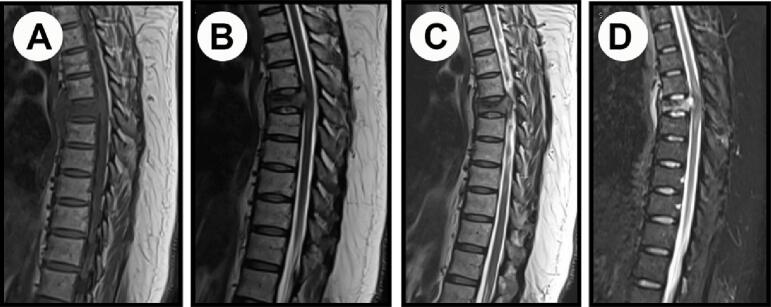
MRI spine of the patient showing a pathological compression fracture and a paravertebral, paravertebral, and retro-vertebral intraspinal mass that is hyperintense on T2 STIR sequence (d) and hypointense on T1 sequence (a), T2 sequence (b), and T2 TSE sequences.

Magnetic resonance imaging (MRI) of the spine revealed a pathological compression fracture of T6 vertebra along with a prevertebral, paravertebral, and retro-vertebral intraspinal mass compressing the spinal cord (Fig. 1). The T6 vertebral body collapsed with a 50 % reduced height with an abnormal T1 hypointense signal and T2/STIR hyperintense signal and heterogenous postcontrast enhancement. There was also noted an element of posterior retropulsion as associated prevertebral/paravertebral and retro-vertebral intraspinal ventral epidural enhancing soft tissue component at the same T5-T6 level with moderate cord compression is seen. Spinal cord intra-medullary edema was visualized with T2 hyperintense signal extending from mid-level of T5 down to the lower level of T7. The disc spaces were intact with normal signal and no pathological enhancement.

**Fig. 2:**
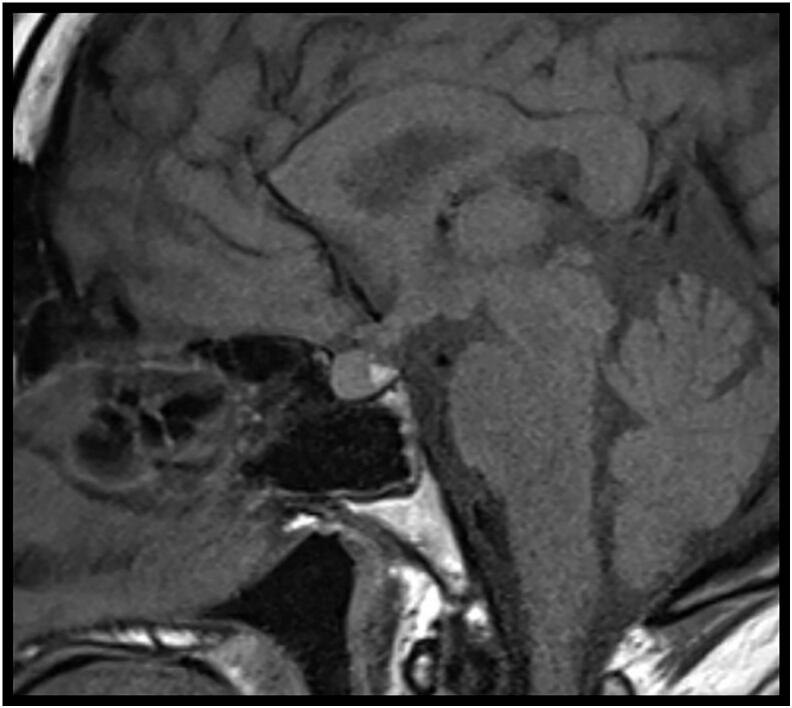
MRI sellar lesion showing pituitary mass with heterogenous signal at T1 sequence with post-contrast enhancement.

Brain MRI was also performed with special focus on sellar region and showed a centralized and thickened pituitary stalk measuring 4 mm in transverse dimensions. The pituitary gland was bulky with a high T1 signal intensity in the posterior lobe (Fig. 2). Mild bilateral cerebral white matter chronic microangiopathic changes were also seen.

Overall, these results found that sellar MRI showed pituitary gland enlargement with hyperintense signal in the posterior lobe. Positron emission tomography (PET)/computed tomography (CT) scan showed an increased uptake in the right parotid gland uptake and renal cortex, indicating asymptomatic pituitary and renal involvement. However the surgical excision, decompression, and screw fixation were performed then.

### 2.4 Surgical procedure

The presence of T6 pathological compression fracture with epidural mass compressing the spinal cord and causing considerable neurological deficits was an indication for surgical intervention. Therefore, the patient underwent T4 to T8 pedicle screws fixation, T6 decompressive laminectomy and biopsy of epidural mass lesion. Following anesthesia, the surgery was performed with the patient laid in prone position of Allen table. Regular prepping and draping were performed, and the surgical level was confirmed with X ray. A skin incision was then made, and the thoracic fascia was opened. Subperiosteal dissection was performed till the corresponding vertebrae were exposed. A free hand technique was used, and pedicle screws were inserted to T4 (4 × 40 Bil), T5 (4 × 40 Bil), T7 (left 4 × 40 Bil, the right side was skipped), and T8 (4 × 40 Bil). An X ray was used to confirm the appropriate location of the screws inserted. T6 laminectomy was then done, and the cord was decompressed. A biopsy was also taken from the ventral epidural soft tissue mass compressing the cord. Ten-millimeter rods were inserted bilaterally, and bone granules with autologous bones chips were applied over the constructs laterally. The homeostasis was maintained, a drain was inserted, the facia was closed with vicryl 2, and the subcutaneous closure was performed using vicryl 0/2. The skin was closed with staplers, and a dressing was applied.

### 2.5 Biopsy and histopathological findings

The specimen sent for pathological examination consisted of two soft and gritty fragments, measuring 0.4 to 0.6 mm, that were entirely submitted in one cassette following decalcification.

The histopathological study of the specimens showed cellular proliferation of oval mononuclear cells characterized by abundant pale cytoplasm, irregular and elongated nuclei with prominent nuclear grooves and folds, fine chromatin and indistinct nucleoli. The cells appeared to infiltrate the bone. Occasional multinucleated giant cells were identified. The background showed mixed inflammatory cell infiltrate composed of neutrophils, eosinophils, plasma cells and lymphocytes. There was no evidence of brisk mitotic activity or necrosis. By immunohistochemical sains, the cells are positive for CD1a, langerin, S100 and CD68. The features were consistent with LCH.

### 2.6 Diagnosis and management plan

The patient was diagnosed as a case of LCH with symptomatic spinal and asymptomatic pituitary involvement. Following surgery, positron emission tomography (PET)/ computed tomography (CT) scan of the spine was performed and showed an increased uptake in the T6 vertebra (likely representing residual disease). There was also noted an increase in the right parotid gland uptake, representing LCH involvement of the gland. An increased uptake was also noted in the renal cortex, indicating resolving acute kidney injury (AKI). Therefore, the patient was recommended to receive few cycles of low intensity systemic chemotherapy such as vinblastine,6-mercaptupurine, or low dose methotrexate.

## 3 Discussion

Vertebral LCH is not uncommonly reported in children [2,8,9]. In adults, spinal involvement is very rare. Only few cases were reported in the literature [5,6]. Thoracic spine is the most common location for vertebral LCH, followed by lumbar and cervical spines [5]. Lesions are usually solitary and involve the vertebral bodies [5].

Langerhans cell histiocytosis of the spine presents with a wide range of clinical symptoms spectrum ranging from asymptomatic to severe neurological deficits due to myelopathy and cord compression [5]. Asymptomatic cases have been reported [1]. Low back pain was the only presentation in other cases, and a wide variation of neurological deficits (e.g., sensory, motor, bowel, or bladder dysfunction) was described in several reported cases [2,5,6,9]. The treatment and prognosis also vary among the cases [10].

Few cases of spinal LCH in adults were reported in the literature, affecting either the cervical, the thoracic, or the lumbar regions. Similar to our case, thoracic LCH was reported in several adult cases in the literature, with different presentations and treatment strategies. For instance, Lim and Hwan [5] reported a 33-year-old man with a continuous enhancing LCH epidural lesion extending from T7 to L1 level and compressing the spinal cord. The patient had also undergone posterior with marginal excision of the epidural mass for spinal cord decompression [5]. He also improved after surgery.

Kouhen et al. [6] have also reported a case of spinal thoracic LCH in an adult 25-year-old male with a long vertebral and epidural lesion extending from T1 to T5. The lesion was symptomatic causing paraparesis, bowel and bladder dysfunction, and sensory deficits [6]. This patient had evidence of systemic involvement in the form of asymptomatic lung LCH, discovered accidently during work-up [6]. His management plan was decompressive irradiation to the dorsal followed by chemotherapy (glucocorticoid and vincristine) [6]. The lesion size regressed following treatment, and his neurological deficits resolved [6].

Kim et al. [11] in 2017, have also reported a case of LCH in the thoracic spine of an adult 45-year-old man who presented with motor and sensory deficits of the lower extremities [11]. His thoracic spine imaging revealed an osteolytic lesion of the eighth and ninth thoracic vertebra causing pathological fracture along with myelopathy from T8 to T10 and cord compression [10]. He was surgically treated with posterior corpectomy and total tumour excision. A mesh cage was inserted, and posterior screw fixation was performed [11]. Following surgery, the patient underwent radiotherapy [11]. He remained stationary for six years during the follow-up without recurrence [11].

Cervical LCH was also reported in adults. Sang-Deok Kim et al. [12] reported a 36-year-old man who presented with neck pain radiating to the upper extremity. His cervical MRI revealed a solitary C5 osteolytic lesion with an enhancing epidural mass compressing the cord, a biopsy of which confirmed the diagnosis of LCH [12]. The patient was successfully treated by C5 anterior cervical corpectomy and fusion with iliac bone graft and had no evidence of tumour recurrence for five years [12].

Fewer cases of LCH in adults were reported to affect the lumbar spine. For instance, Feng et al. [13] in 2012, described a 51-year-old man who presented with low back pain, weakness of the trunk muscles, and lower extremity numbness. His spinal imaging revealed a single osteolytic lesion at the fourth lumbar vertebra with cord compression [13]. The histopathology results confirmed the diagnosis of LCH, and a systemic screen was performed and excluded any systemic involvement [13]. This patient was treated by percutaneous vertebroplasty, which is an alternative treatment that stands mid-way between invasive overdue surgical treatment and conservate, relatively inadequate, medical treatment [13]. Following percutaneous vertebroplasty, the patient received three cycles of chemotherapy and remained stable for six months without evidence of recurrence [13]. Other closely similar cases were reported by Kevane et al. [14] and Cordon et al. [15] where patients with spinal LCH were successfully treated by percutaneous vertebroplasty with good clinical result.

Hassan et al. [10] also reported a case of lumbar LCH in an adult. He was a 35-year-old man who presented solely with low back pain without neurological deficits [10]. His imaging revealed an osteolytic lesion at L1 vertebra with no considerable compression on the spinal cord [10]. He was conservatively managed and followed for two years with no evidence of recurrence [10].

As discussed, different treatment strategies exist in the literature for spinal LCH. To date, there are no guidelines or consensus recommendations for the optimal treatment strategy for spinal LCH [5]. Treatment approaches include conservative management (i.e., bed rest, bracing, cast and immobilization), surgery, chemotherapy, or radiation therapy. Surgery is usually preferred in patients with spinal cord compression, like our case presented here, to improve functional recovery and to obtain a biopsy for histopathological study [5]. Chemotherapy may be the first line treatment of choice for patients with no neurological dysfunction [11]. It can also be used as an adjuvant therapy following surgical decompression, particularly in patients with systemic involvement (such as our described patient) or in patients with multiple spinal lesions [5].

The prognosis of spinal LCH is also variable based on the clinical presentation, number and sites of lesions, systemic involvement, and patient's characteristics [11]. In patients with solitary spinal LCH, the prognosis is usually good, and spontaneous resolution is seen in a considerable proportion of patients within one or two years. The prognosis of patients with multiple lesions, or extensive systemic involvement is poor [1,3].

## Patient consent

Written informed consent was obtained from the patient for the publication of this case report and accompanying images. A copy of the written consent is available for review by the Editor-in-Chief of this journal on request.

## Ethical approval

Baghdad hospital exempts ethical approval and supply the informed written consent for this case report.

## Funding

There were no sponsors for this case report.

## Research registration number

N/A.

## CRediT authorship contribution statement

All authors equally contributed to the analysis and writing of the manuscript.

## Guarantor

Md Moshiur Rahman.

## Declaration of competing interest

The authors declare that they have no known competing financial interests or personal relationships that could have appeared to influence the work reported in this paper.

## References

[1] Tillotson C.V., Anjum F., Patel B.C. (2022). Langerhans cell histiocytosis. https://www.ncbi.nlm.nih.gov/books/NBK430885/.

[2] Su M., Gao Y.J., Pan C., Chen J., Tang J.Y. (2018). Outcome of children with Langerhans cell histiocytosis and single-system involvement: a retrospective study at a single center in Shanghai, China. Pediatr. Hematol. Oncol..

[3] Jha S.K., de Jesus O. (2022). Eosinophilic Granuloma. https://www.ncbi.nlm.nih.gov/books/NBK559038/.

[4] Kuttner B.J., Friedman K.J., Burton C.S., Olsen E.A. (1987). Letterer-Siwe disease in an adult. Cutis.

[5] Lim C.S., Cho J.H. (2020). Spinal epidural involvement in adult Langerhans cell histiocytosis (LCH): a case report. Medicine (United States).

[6] Kouhen F., Benhmiddou N., Afif M. (2015). Adult Langerhans cell histiocytosis: a rare etiology of spinal cord compression. Open J. Intern. Med..

[7] Agha R.A., Franchi T., Sohrab C., Mathew G., Kirwan A., Thomas A. (2020). The SCARE 2020 guideline: updating consensus surgical case report (SCARE) guidelines. Int. J. Surg..

[8] Sadashiva N., Rajalakshmi P., Mahadevan A., Vazhayil V., Rao K.N., Somanna S. (2016). Surgical treatment of Langerhans cell histiocytosis of cervical spine: case report and review of literature. Childs Nerv. Syst..

[9] Peng X.S., Pan T., Chen L.Y., Huang G., Wang J. (2009). Langerhans’ cell histiocytosis of the spine in children with soft tissue extension and chemotherapy. Int. Orthop..

[10] Hassan B.W., Moon B.J., Kim Y.J., Kim S.D., Choi K.Y., Lee J.K. (2016). Langerhans cell histiocytosis in the adult lumbar spine: case report. Springerplus..

[11] Kim M.C., Sung S.H., Cho Y. (2017). Langerhans cell histiocytosis of the thoracic spine in an adult. Korean J. Spine.

[12] Kim S.D., Moon B.J., Choi K.Y., Lee J.K. (2017). Primary Langerhans cell histiocytosis (LCH) in the adult cervical spine: a case report and review of the literature. Interdiscip. Neurosurg..

[13] Feng F., Tang H., Chen H., Jia P., Bao L., Li J.J. (2013). Percutaneous vertebroplasty for Langerhans cell histiocytosis of the lumbar spine in an adult: case report and review of the literature. Exp. Ther. Med..

[14] Percutaneous vertebroplasty in osteoporosis, myeloma and Langerhans' cell histiocytosis - PubMed. https://pubmed.ncbi.nlm.nih.gov/19772001/.

[15] Cardon T., Hachulla E., Flipo R.M. (1994). Percutaneous vertebroplasty with acrylic cement in the treatment of a Langerhans cell vertebral histiocytosis. Clin. Rheumatol..

